# Clinical evaluation of SafeCEC^®^ one-way valve in hemolysis during CPB: Pilot study[Fn FN1]

**DOI:** 10.1051/ject/2025010

**Published:** 2025-06-16

**Authors:** Henrique Madureira da Rocha Coutinho, Joaquim Henrique de Souza Aguiar Coutinho, José Hamilton Torres, Rafaela Mourão Casanova, Gustavo Kikuta, Edison Emídio Reis

**Affiliations:** 1 Division of Cardiovascular Surgery of the Hospital Universitário Pedro Ernesto – UERJ Rua Boulevard 28 de Setembro, 77 – Vila Isabel Rio de Janeiro RJ 20551-030 Brazil; 2 Division of Cardiovascular Surgery of the Hospital das Clínicas de Teresópolis Costantino Ottaviano – UNIFESO Avenida Alberto Torres, 111 – Alto Teresópolis RJ 25964-004 Brazil

**Keywords:** Cardiopulmonary bypass, One-way valve, Centrifugal pump, Retrograde flow

## Abstract

*Introduction*: In cardiopulmonary bypass (CPB), blood circulation is temporarily maintained by an artificial blood-pumping device during cardiac surgery. Worldwide, approximately half of the CPB procedures utilize either centrifugal or roller pumps (Wu P et al. Front Physiol 12, 699891). Centrifugal pumps, while non-occlusive, pose a risk of blood reflux if there is a system failure, which endangers patient safety (Souza MHL, Elias DO. Fundamentos da Circulação Extracorpórea 2006; 186–192). SafeCEC^®^, a one-way valve, offers a potential solution to this risk by preventing arterial line reflux. This pilot study aims to evaluate patient safety by analyzing hemolysis as an evaluation parameter. Plasma free hemoglobin is chosen to measure patient safety with the use of the product, ensuring it does not cause additional hemolysis during extracorporeal circulation. *Materials and methods*: After approval by the Ethics Committee, 31 patients undergoing CPB with a centrifugal pump were included in the study. The patients were randomly divided into two groups: group A, where SafeCEC^®^ was incorporated into the arterial line, and group B, which used the conventional circuit. Hemolysis was assessed by analyzing plasma free hemoglobin in blood samples collected before CPB, after CPB, and 24 h after weaning from CPB. *Results*: This device has been shown to be effective in controlling blood reflux, eliminating the need for arterial line clamps. Analysis of plasma free hemoglobin levels revealed no significant differences between the groups with or without SafeCEC^®^. *Conclusion*: The SafeCEC^®^ one-way valve effectively prevents reflux without contributing to blood damage, as indicated by the absence of significant hemolysis. This pilot study demonstrates that the SafeCEC^®^ is both safe and effective for its intended use.

## Introduction

In cardiopulmonary bypass (CPB), blood circulation is artificially driven by a blood pumping device that temporarily replaces cardiac function by maintaining blood circulation during cardioplegic arrest [1]. Currently, centrifugal pumps are widely used for CPB due to their non-occlusive mechanism, which reduces the risk of excessive hemolysis and high afterload [2]. However, for procedures lasting less than 3 h, there is no conclusive evidence demonstrating their superiority over roller pumps, which maintain consistent flow and pressure but may be associated with higher hemolysis rates due to their occlusive nature [3, 4].

Centrifugal pumps are characterized by a non-occlusive nature and sensitivity to flow resistance, that is, the flow generated by the pump varies inversely with variation of flow resistance; if the resistance is equal to the outlet pressure of the pump, no flow occurs. Conversely, if the flow resistance is greater than the pump outlet pressure, retrograde flow will occur [5], which may result in blood volume loss, hypotension, surgical exsanguination of the patient and other consequences if the event is not immediately detected and controlled [6]. Retrograde flow is a serious risk to patient safety without an anti-reflux valve.

Retrograde flow detection in centrifugal pumps is performed through continuous monitoring of flowmeter, but the control of retrograde flow depends solely on the actions of the perfusionist, who must occlude the line through clamping to avoid reflux by accelerating the rotation the centrifugal pump drive unit until it reaches 1200–1500 rpm [7]. At this point, the perfusionist must end the flow. Likewise, in weaning form CPB, the arterial line must be clamped while the pump still rotates.

These are the product usage instructions that the manufacturers of such pumps have defined to prevent retrograde blood flow in the arterial line. These instructions mandate the establishment and maintenance of a minimum rotation speed exceeding the patient’s resistance, with the arterial line clamped before halting the pump’s rotation [8]. This method imposes mechanical stress on the blood, that may be harmful [9]. The perfusionist undertakes to initiate or cease blood flow at rotation levels higher than necessary, potentially increasing trauma to blood cells and contributing to increase hemolysis in CPB.

Hemolysis is defined as the rupture of red blood cells, leading to the release of its contents, such as hemoglobin and lactate dehydrogenase, into the plasma. The hemoglobin released in plasma, or plasma free hemoglobin, subsequently binds to the circulating haptoglobins, which are then metabolized in the liver. However, when hemoglobin release exceeds the plasma haptoglobin concentration, plasma free hemoglobin will exert its deleterious effects causing complications such as acute kidney injury [10].

AmSECT, the American Society of ExtraCorporeal Technology, as well as the Society of Clinical Perfusion Scientists, the Society for Cardiothoracic Surgery, the Association for Cardiothoracic Anaesthesia and Critical Care of Great Britain & Ireland, and Brazilian Society for Extracorporeal Circulation, together with Brazilian Society of Cardiovascular Surgery, recommend that at least one method be used to prevent retrograde blood flow during CPB in circuits using centrifugal pumps [11, 12]. This document recommends that at least one method of preventing backflow when using a centrifugal pump should be used during CPB procedures, such as one-way flow valves; hard-stop check controls to prevent accidental reduction in pump speed; electronically activated arterial line clamps; or a low-speed visual and audible alarm [11].

**Figure 1:**
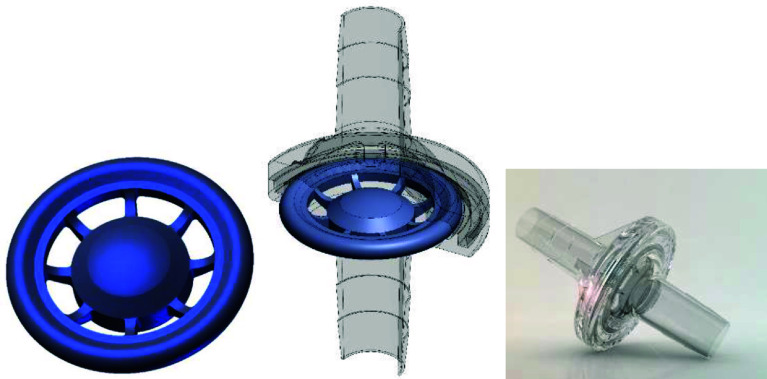
A: detailed depiction of the valve design; a central plug connected by rods equidistant to an outer circumference sized to equalize the line passage area, resulting in a smaller rod passage area and inlet and outlet connections 3/8”. This design, resembling a cartwheel, inspired the piece’s name. B: SafeCEC^®^ drawing showing the housing profile. C: Illustrative image from SafeCEC.

In Brazil, addressing this issue constitutes a significant aspect of a perfusionist’s daily responsibilities, given the absence of available devices to meet this requirement. The SafeCEC^®^ valve is constructed of silicone and affixed to polycarbonate inlet and outlet connectors with a 3/8″ diameter. It is positioned in the arterial line of the CPB circuit, specifically in the segment after the centrifugal pump outlet. This valve’s primary feature is its capability to permit flow in a unique direction. It was designed in the form of a cartwheel [13, 14], with a central plug fixed to the center by slender rods attached to the outer rim. As flow initiates and pressure builds at the valve inlet, the valve plug moves away from the inlet port, allowing flow towards the valve outlet. By ceasing flow at the inlet, the valve closes, preventing backflow from occurring (Fig. 1).

The SafeCEC^®^ valve is constructed of silicone and affixed to polycarbonate inlet and outlet connectors. Positioned within the blood pump outlet line of the CPB circuit, this valve’s primary feature is the ability to permit flow in a singular direction. It was designed in the form of a cartwheel, that is, a central plug fixed to the center by slender rods attached to the outer rim. As flow initiates and pressure builds at the valve inlet, the valve plug moves away from the inlet port, allowing flow towards the valve outlet. By ceasing flow at the inlet, the valve closes, preventing backflow from occurring.

To clinically evaluate SafeCEC^®^, we propose a pilot randomized comparative study to assess its safety and efficacy in cardiopulmonary bypass. Our objective includes verifying whether the use of SafeCEC^®^ increases patient risk, comparing the degree of blood trauma produced in CPB procedures with and without the use of SafeCEC^®^.

## Materials and methods

After approval by the Ethics Committee of Hospital Pedro Ernesto – Universidade do Estado do Rio de Janeiro (Rio de Janeiro, Brazil), 31 patients who underwent CPB with a centrifugal pump were included. Patients were randomly assigned using the random function in Microsoft Excel, without blinding, and divided into two groups: group A, in which SafeCEC^®^ was incorporated into the arterial line, and group B, where the conventional circuit was used. Inclusion criteria of patients over 18 years of age, with indication for elective cardiac surgery with CPB, who agreed to participate in this study.

Hemolysis was evaluated using the following protocol: blood samples were collected from each participant for analysis of plasma free hemoglobin, before CPB, after CPB, and 24 h after weaning of CPB. Plasma free hemoglobin analysis was performed by collecting samples and subjecting them to centrifugation for 10 min at 1500 rpm. Subsequently, 1 mL of plasma was collected, centrifuged again at 1500 rpm and analyzed by HemoCue^®^Plasma/Low Hb system as per its instructions for use.

Other parameters analyzed were arterial blood flow, CPB time, diagnosis, type of surgery performed, hematocrit levels, and descriptive data. Exploratory data analysis comprised the calculation of descriptive statistics, including measures of central tendency (mean), dispersion (standard deviation), percentiles, and the minimum and maximum values for numerical variables, alongside frequencies and proportions for categorical variables. To evaluate the distributional properties of continuous variables, the Shapiro-Wilk test was employed to assess adherence to the assumption of normality [15].

For comparisons of continuous variables between two independent groups, parametric data were analyzed using the Student’s *t*-test. In instances where the assumption of homogeneity of variances was violated, Welch’s correction was applied to ensure the robustness of the inference. Effect sizes were quantified using Cohen’s *d*, a widely recognized measure of effect magnitude, interpreted as follows: 0.2 representing a small effect, 0.5 a medium effect, and 0.8 or higher a large effect [16].

For non-parametric data, the Mann-Whitney *U*-test was utilized to compare scale scores across independent variables. In these analyses, the effect size was reported as the point-biserial correlation coefficient (*r*), which expresses the strength of association between a dichotomous and a continuous variable. Interpretation of *r* followed the thresholds proposed by Cohen [17], with 0.10–0.29 representing a small effect, 0.30–0.49 a medium effect, and values above 0.50 indicating a large effect.

Associations between categorical variables were examined using the chi-square test of independence. Effect sizes for these associations were calculated using the Phi coefficient, which ranges from 0 to 1, with values between 0.10 and 0.29 denoting a small effect, 0.30 to 0.49 a medium effect, and 0.50 or greater indicating a large effect [18].

All statistical analyses were conducted using IBM SPSS Statistics version 29 (IBM Corporation, Armonk, NY, 113 USA) and Jamovi software (The Jamovi Project, 2023). A significance threshold of *p* < 0.05 was applied throughout [19]. We did not use the *t*-test because the hemoglobin data did not have a normal distribution; and with a small sample size, it was even more important to use other tests, in this case, non-parametric tests, which is what we did, using the Mann-Whitney test. Pilot studies do not aim to have high power to detect differences, but rather to assess the feasibility of the research, so that a larger study can be conducted later.

## Results

The results of this study showed that the use of SafeCEC^®^ in CPB with a centrifugal pump did not have a significant impact on hemolysis, compared to the conventional circuit. The perfusionist routinely uses clamps to close the arterial line when operating the heart-lung machine. With the use of SafeCEC^®^, it was observed that it was not necessary to use clamps, because when the perfusionist reduced the pump rotation and consequently, the blood flow, the valve acted immediately, preventing the occurrence of reflux and without the need to clamp the arterial line. This increases the feeling of safety and eliminates a task for the perfusionist in the CPB operation.

Exploratory data analysis included the descriptive statistics mean and standard deviation. To analyze the behavior of continuous variables, descriptive statistics and the Shapiro-Wilk test for normality assumption were considered. Among the 31 patients recruited, 22 were male (71%) and 9 were female (29%). The mean age of this study population was 59.9 ± 12.4 years (Table 1). The mean CPB time was 102.8 ± 40.5 min, and the mean aortic clamping time was 80.33 ± 33 min (Table 2).

**Table 1:** Summary of demographic data for study participants.

Variables	Statistics
Age	59.9 ± 12.4 years
Gender, *n* (%)	
Female	9 (29.0)
Male	22 (71.0)

Categorical variables are described as numbers (percentages); continuous variables are described as mean ± standard deviation.

**Table 2:** Summary of descriptive findings.

Variables	Mean ± standard deviation	Minimum	Percentile			Maximum
			25	50	75	
Age	59.9 ± 12.4 years	23	55	58	70	81
Arterial flow	5.16 ± 0.50 L/min	3.7	4.9	5.1	5.4	6.2
CPB time	102.8 ± 40.5 min	56	69	85	127	200
Aortic clamping time	80 ± 33 min	42	59.2	69	91.25	171
Initial hemoglobin level	10.57 ± 2.49 mg/dL	5.2	9.3	10.3	12.5	16.3
Final hemoglobin level	10 ± 4 mg/dL	5.7	8.3	9.8	10.2	30.6
Initial hematocrit level	31.48 ± 8.5%	19	28	32	37	48
Final hematocrit level	28.68 ± 4.7%	17	25	30	31	38
Plasma free hemoglobin before CPB	0.02 ± 0.17 mg/dL	0.01	0.01	0.02	0.02	0.09
Plasma free hemoglobin after CPB	0.08 ± 0.48 mg/dL	0.02	0.037	0.075	0.10	0.21
Plasma free hemoglobin 24 h after CPB	0.03 ± 0.15 mg/dL	0.01	0.02	0.02	0.04	0.07

Blood samples for analysis of hemoglobin, hematocrit and free plasma hemoglobin were collected before CPB, at the end of CPB and 24 h post-CPB.

The mean baseline plasma free hemoglobin before CPB was 0.02 ± 0.17 mg/dL and after CBP was 0.08 ± 0.48 mg/dL, with a mean variation of 0.06 mg during CPB. However, after 24 h, levels returned to baseline. To compare continuous variables between the two groups, Student’s *t*-test was employed for parametric data. In cases where the data lacked homogeneity of variance, Welch’s correction was used to interpret the results [16]. Cohen’s *d* was used as the effect size, offering a statistical measure to quantify the difference between two groups in terms of standard deviations. Regarding the type of surgery performed, the vast majority of the studied population (70.9%) underwent coronary artery bypass grafting surgeries, alone or combined with other procedures. Table 3 shows the categorical variables related to surgery.

**Table 3:** Summary of surgical procedures.

Variables	Statistics
	*n* (%)
Mitral valve replacement	
No	26 (83.9)
Yes	5 (16.1)
Tricuspid valve repair	
No	30 (96.8)
Yes	1 (3.2)
Aneurysm repair	
No	29 (93.5)
Yes	2 (6.5)
Closure of interatrial communication	
No	30 (96.8)
Yes	1 (3.2)
Coronary artery bypass grafting	
No	9 (28.8)
Yes	22 (70.9)
Aortic valve replacement	
No	10 (32.3)
Yes	21 (67.7)

Categorical variables are described as numbers (percentages).

### Bivariate analysis of demographic and clinical variables by group

According to Table 4, there was no significant difference in hemolysis or plasma free hemoglobin concentration between groups. In group A, Group A (SafeCEC), the mean plasma hemoglobin after CPB was 0.08 ± 0.05 versus 156 0.07 ± 0.05 in group B. Group A had two indications for blood transfusion versus three indications in group B. There was one death in group A, but the cause was unrelated to the use of the SafeCEC^®^ valve and plasma hemoglobin data were not recorded. No adverse events were reported in the study population.

**Table 4:** Comparative analyses of clinical variables by group.

Variable	Group	*N*	Mean ± SD	*P*-value	Effect size
CPB time	A	15	111.1 ± 44.3	0.439⸸	0.167
	B	16	95 ± 36.3		
Aortic clamping time	A	14	83.6 ± 33.1	0.506⸸	0.147
	B	16	76.9 ± 33.8		
Initial hemoglobin level	A	15	10.4 ± 1.8	0.419*	−0.295
	B	16	11.1 ± 3		
Final hemoglobin level	A	15	10.8 ± 5.5	0.937⸸	0.021
	B	16	9.5 ± 2		
Initial hematocrit level	A	15	29.8 ± 7.8	0.293*	−0.385
	B	16	33.1 ± 9.1		
Final hematocrit level	A	15	28 ± 2.5	0.443*^W^	−0.278
	B	16	29.3 ± 6.2		
Plasma free hemoglobin before CPB	A	14	0.02 ± 0.01	0.5⸸	0.138
	B	16	0.02 ± 0.02		
Plasma free hemoglobin after CPB	A	14	0.08 ± 0.05	0.451⸸	0.165
	B	16	0.07 ± 0.05		
Plasma free hemoglobin 24 h after CPB	A	14	0.03 ± 0.02	0.201⸸	0.263
	B	16	0.02 ± 0.01		

SD: standard deviation; ES: effect size: Mann-Whitney test (effect size: *r* biserial point). *Student’s *t*-test (Cohen’s *d*); ^w^Welch’s correction; ⸸Welch’s correction was applied due to violation of homogeneity of variances.

The effect size used in the comparison of continuous variables, such as age tested by Student’s *t*-test, was Cohen’s *d*. This statistical measure can be used to quantify the magnitude of the difference between two groups in terms of standard deviations. Interpretation of Cohen’s *d* is as follows: 0.2 denotes a small effect, 0.5 indicates a medium effect, and 0.8, a large effect [17]. Therefore, the effect size obtained in the analysis confirms the absence of age difference between the two groups.

The association between group and gender was analyzed using the chi-square test of independence. Phi was employed to calculate the effect size, ranging from 0 to 1, with higher values indicating a stronger association; therefore, as can be seen, the effect size was negligible.

As mentioned in Materials and methods section, the effect size used in the comparisons by the Mann-Whitney test was the *r* biserial point. This measure of effect size indicates the strength of association between a dichotomous variable and a continuous variable. Like other correlation values, its range spans from −1 to 1, with values closer to 1 indicating a stronger association. According to Cohen [14], the cut-off points for interpretation are as follows: small: *r* = 0.10 to 0.29; medium: *r* = 0.30 to 0.49; large: *r* = 0.50 and above.

No significant differences were observed for the variables tested in Table 5; in fact, the effect size for all variables was small.

**Table 5:** Comparative analyses of clinical variables by group.

Variable	Group	*N*	Mean ± SD	*P*-value	Effect size
CPB time	A	15	111.1 ± 44.3	0.439⸸	0.167
	B	16	95 ± 36.3		
Aortic clamping time	A	14	83.6 ± 33.1	0.506⸸	0.147
	B	16	76.9 ± 33.8		
Initial hemoglobin level	A	15	10.4 ± 1.8	0.419*	−0.295
	B	16	11.1 ± 3		
Final hemoglobin level	A	15	10.8 ± 5.5	0.937⸸	0.021
	B	16	9.5 ± 2		
Initial hematocrit level	A	15	29.8 ± 7.8	0.293*	−0.385
	B	16	33.1 ± 9.1		
Final hematocrit level	A	15	28 ± 2.5	0.443*^W^	−0.278
	B	16	29.3 ± 6.2		
Plasma free hemoglobin before CPB	A	14	0.02 ± 0.01	0.5⸸	0.138
	B	16	0.02 ± 0.02		
Plasma free hemoglobin after CPB	A	14	0.08 ± 0.05	0.451⸸	0.165
	B	16	0.07 ± 0.05		
Plasma free hemoglobin 24 h after CPB	A	14	0.03 ± 0.02	0.201⸸	0.263
	B	16	0.02 ± 0.01		

SD: standard deviation; ES: effect size: Mann-Whitney test (effect size: *r* biserial point). *Student’s *t*-test (Cohen’s *d*); ^w^Welch’s correction; ⸸Welch’s correction was applied due to violation of homogeneity of variances.

**Figure 2:**
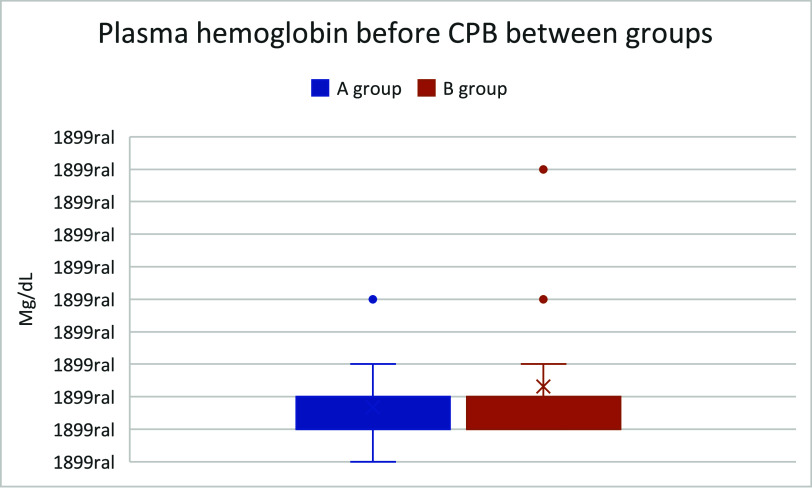
Boxplot comparing plasma hemoglobin before CPB between groups.

**Figure 3:**
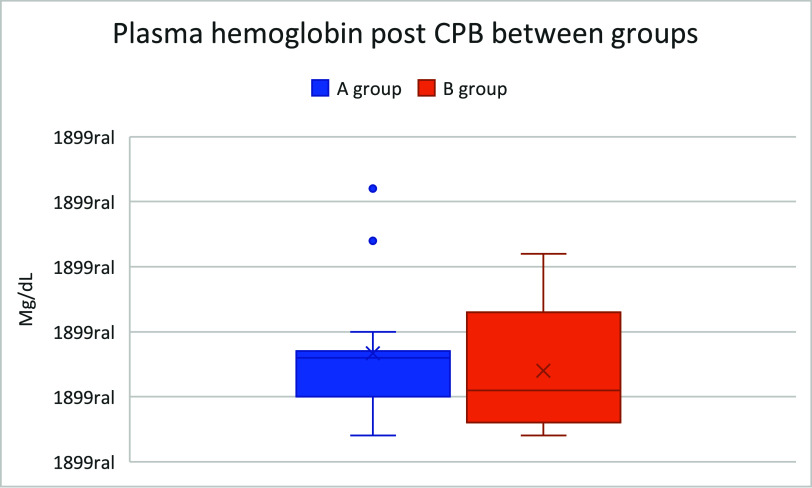
Boxplot comparing plasma hemoglobin after CPB between groups.

**Figure 4:**
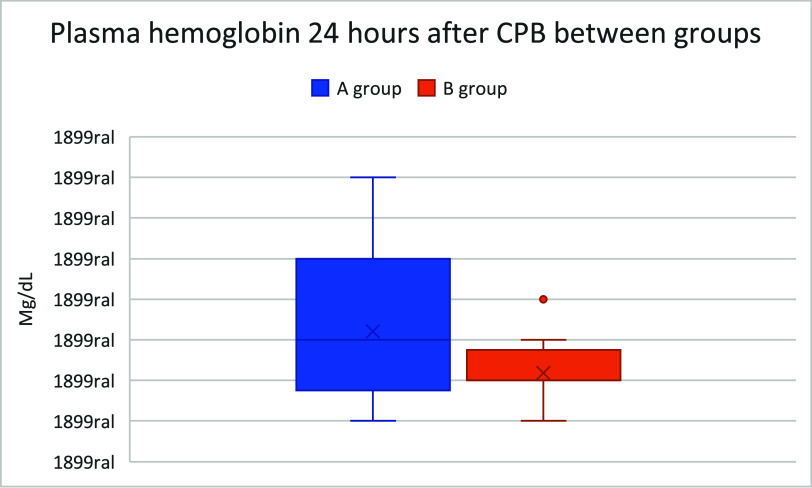
Boxplot comparing plasma hemoglobin 24 h after CPB between groups.

The boxplots presented in Figures 2–4 illustrate the distribution of hemolysis markers across the study groups. Notably, Figure 2 includes extreme outliers that significantly expand its scale compared to Figures 1 and 3. To preserve the integrity of each dataset, the *Y*-axis scales were not standardized across figures, as doing so would artificially compress the distribution of Figures 1 and 3, making it difficult to discern variability and quartile distribution in these groups.

This visualization approach ensures that the true dispersion and data trends remain accurately represented, preventing misinterpretation of hemolysis levels. While some outliers in Figure 2 suggest increased variability, they do not affect the median and interquartile range observed in the other groups. Future studies may explore alternative statistical normalization techniques; however, in this analysis, retaining distinct scales provides a clearer and more clinically relevant interpretation of SafeCEC^®^ performance in preventing retrograde flow-induced hemolysis.

The results of this study suggest that the use of SafeCEC^®^ in CPB with a centrifugal pump is safe and does not elevate the hemolysis normally generated by CPB.

## Discussion

Hemolysis is found in all surgical procedures using extracorporeal circuits. Several studies have identified rising levels of plasma free hemoglobin [18, 19], designating it as a marker of blood trauma. Hemolysis can occur in three distinct ways: natural selection of the spleen, physicochemical imbalance [20], or by exposing cells to mechanical stress conditions [21], as observed during artificial blood pumping [22].

In the case of CPB, hemolysis occurs by mechanical stress, induced either by direct trauma from blood passage through rollers or exposure to different surfaces at different speeds. Centrifugal pumps, favored by Brazilian perfusionists, offer an alternative to roller pumps. Thus, measurement of hemolysis serves as a sensitive means to assess the safety of a medical device added into the arterial line of the CPB circuit.

Evaluation with pre-CPB, post-CPB and 24-hour measurements is an appropriate method for the clinical evaluation of SafeCEC^®^.

According to the article Estatística Cardiovascular – Brasil 2021, published in the journal *Arquivos Brasileiros de Cardiologia*, in 2019, 79,590 cardiac surgeries were performed in Brazil, with 46,362 (58.2%) involving CPB and 33,228 (41.8%) without CPB [18]. We estimate that 30–40% of surgeries with CPB have used a centrifugal pump, equating to 15,000–18,000 surgeries, or possibly even a higher number [23]. This shows the significant exposure to the risk of blood reflux associated with the use of a centrifugal pump in case of mechanical failure or delay in clamping the arterial line.

The uncertainty surrounding the safety of centrifugal pumps has led organizations such as the American Society of ExtraCorporeal Technology, the Society of Clinical Perfusion Scientists, the Society for Cardiothoracic Surgery and the Association for Cardiothoracic Anaesthesia and Critical Care of Great Britain & Ireland to recommend the adoption of at least one method to mitigate the risk of retrograde blood flow during CPB in circuits using centrifugal pumps. Standard 6.7 from AMSECT Guideline (Minneapolis, MN) [11] stipulates that “At least one method must be used to prevent retrograde flow for systemic circulation in circuits with centrifugal pumps”. The document also cites one-way flow valves as examples of safety devices for controlling backflow. According to Kolff et al. [24], reflux and the resulting negative pressure act as a hemodynamic siphon, aspirating the patient’s arterial blood and potentially leading to patient exsanguination and its consequences. In addition, according to Kolff et al. [6], a reflux can start within just 540 milliseconds after stopping the pump and this reflux can reach a flow rate of 2.5 L/min after another 470 milliseconds.

In this pilot study, SafeCEC^®^ demonstrated effectiveness in controlling reflux in the arterial line. Its operation eliminates the need for arterial line clamping, enhancing the safety and ease of CPB initiation and termination for the perfusionist. The valve stops the flow when the pressure at the inlet equals the outlet pressure, preventing backflow. In this way, SafeCEC^®^ fulfills its objective of protecting the arterial line from reflux without causing an increase in hemolysis during CPB. *This valve reduces the immediate need for arterial line clamping upon coming off CPB, thereby simplifying the transition process for the perfusionist. However, appropriate clamping remains necessary at a later stage to ensure optimal procedural safety, particularly if the centrifugal pump drive is off and there is no concern for shear stress on blood cells during clamping.* In addition, the SafeCEC^®^ valve did not cause additional damage to the blood, as evidenced by the hematological and biochemical parameters evaluated.

There was no significant difference between the groups with and without the SafeCEC^®^ valve.

## Conclusion

The perfusionist, responsible for conducting CPB procedures, is aware of the risks, human factors and mechanical failures that can occur during CPB. In each case, the goal is to provide a safe and uneventful perfusion process. Safe perfusion entails understanding and implementation of best practices for CPB procedures, adequate training and well-defined protocols and instructions for handling various controls for conducting a CPB procedure. Additionally, continuous monitoring of vital functions is imperative to maintain patient safety throughout CPB [25].

The SafeCEC^®^ one-way valve adequately fulfills this requirement. However, this study is limited by its small sample size and single-center design. To calculate the sample size, an expected effect size of 0.50, a study power of 80% and a significance level of 5% were considered. This calculation yielded a requirement of 67 patients in each group, totaling 134 patients in the sample.

Given these limitations, we recommend that future studies be carried out with a larger number of patients, across different centers, with a control group without the valve, and incorporating a wider range of clinical outcomes and a cost-effectiveness analysis.

## Data Availability

The datasets generated and analyzed during the current study are available from the corresponding author upon reasonable request.
